# Physical activity, sedentary behaviors and dietary habits among Saudi adolescents relative to age, gender and region

**DOI:** 10.1186/1479-5868-8-140

**Published:** 2011-12-21

**Authors:** Hazzaa M Al-Hazzaa, Nada A Abahussain, Hana I Al-Sobayel, Dina M Qahwaji, Abdulrahman O Musaiger

**Affiliations:** 1 Director of Exercise Physiology Laboratory, Department of PE and Movement Sciences, College of Education, King Saud University, Riyadh, Saudi Arabia; 2 Scientific Boards, Obesity Research Chair, King Saud University, Riyadh, Saudi Arabia; 3 Director of School Health, Ministry of Education, Eastern Province, Saudi Arabia; 4 Department of Rehabilitation Sciences, College of Applied Medical Sciences, King Saud University, Riyadh, Saudi Arabia; 5 Department of Clinical Nutrition, College of Applied Medical Sciences, King Abdulaziz University, Jeddah, Saudi Arabia; 6 Director, Arab Center for Nutrition, Manama, Bahrain, and Nutrition and Health Studies Unit, Deanship of Scientific Research, University of Bahrain

**Keywords:** Physical activity, sedentary behaviors, dietary habits, lifestyle factors, adolescents, Saudi Arabia

## Abstract

**Background:**

Few lifestyle factors have been simultaneously studied and reported for Saudi adolescents. Therefore, the purpose of the present study was to report on the prevalence of physical activity, sedentary behaviors and dietary habits among Saudi adolescents and to examine the interrelationships among these factors using representative samples drawn from three major cities in Saudi Arabia.

**Methods:**

This school-based cross-sectional study was conducted during the years 2009-2010 in three cities: Al-Khobar, Jeddah and Riyadh. The participants were 2908 secondary-school males (1401) and females (1507) aged 14-19 years, randomly selected using a multistage stratified sampling technique. Measurements included weight, height, sedentary behaviors (TV viewing, playing video games and computer use), physical activity using a validated questionnaire and dietary habits.

**Results:**

A very high proportion (84% for males and 91.2% for females) of Saudi adolescents spent more than 2 hours on screen time daily and almost half of the males and three-quarters of the females did not meet daily physical activity guidelines. The majority of adolescents did not have a daily intake of breakfast, fruit, vegetables and milk. Females were significantly (*p *< 0.05) more sedentary, much less physically active, especially with vigorous physical activity, and there were fewer days per week when they consumed breakfast, fruit, milk and diary products, sugar-sweetened drinks, fast foods and energy drinks than did males. However, the females' intake of French fries and potato chips, cakes and donuts, and candy and chocolate was significantly (*p *< 0.05) higher than the males'. Screen time was significantly (*p *< 0.05) correlated inversely with the intake of breakfast, vegetables and fruit. Physical activity had a significant (*p *< 0.05) positive relationship with fruit and vegetable intake but not with sedentary behaviors.

**Conclusions:**

The high prevalence of sedentary behaviors, physical inactivity and unhealthy dietary habits among Saudi adolescents is a major public health concern. There is an urgent need for national policy promoting active living and healthy eating and reducing sedentary behaviors among children and adolescents in Saudi Arabia.

## Background

Globally, noncommunicable diseases (NCDs) are the leading causes of death, killing more people each year than all other causes combined [1]. According to the World Health Organization (WHO) Report 2002, the most important risks of NCDs included high blood pressure, high concentrations of cholesterol in the blood, inadequate intake of fruit and vegetables, being overweight or obese, physical inactivity and tobacco use [2]. Five of these risk factors are closely related to diet and physical activity. Thus, it is well recognized that diet and physical activity play important roles in maintaining health and preventing diseases [3]. Findings from a recent cardiovascular risk study among young Finns confirmed that dietary patterns have a role in the development of cardiovascular disease [4].

At present, there is a real concern about the increase of unhealthy dietary habits, including skipping breakfast and greater consumption of sweetened soft drinks by young people, and the possible role of these habits in the pathogenesis of childhood obesity [5-7]. Data from a study conducted on American adolescents indicated that breakfast consumption during school years was associated with about a 30% lower likelihood of later becoming overweight or obese [8]. Moreover, research studies and reviews indicate that breakfast skipping is highly prevalent among adolescents in the United States and Europe [9] as well as in many Arab countries [10-12].

Participation in health-enhancing physical activity is a key determinant of energy expenditure in youth and leads to improved cardiovascular and metabolic fitness as well as enhanced bone health [13-16]. Persistent physical inactivity, on the other hand, is detrimental to health and well-being [3,17], and it was shown to be associated with a less healthy lifestyle [18].

Furthermore, sedentary behaviors--a subject that has recently undergone intense research--are believed to be associated with adverse health outcomes in a way that is thought to be different from those attributed to lack of physical activity [17]. Indeed, recent research findings have shown that television (TV) viewing (sedentary activity) and physical activity appear to be separate entities and are independently associated with obesity and metabolic risk [19]. In another study, sedentary behaviors and physical activity were shown to be separately linked to distinct dietary habits: physical activity appears to be associated more with healthy choices, while sedentary activities are related more to unhealthy choices [20]. Findings from a longitudinal study indicated that changes in TV viewing were not associated with changes in leisure-time moderate-to-vigorous physical activity among 10- to 15-year-old adolescents, and it was concluded that the two behaviors represent two separate constructs, not functional opposites [21]. What is most alarming is the clustering effect of such unhealthy lifestyle habits among children and adolescents. Results of a systematic review indicated that sedentary behaviors, as measured by total screen-viewing time, were clearly associated with lower fruit and vegetable intake and higher consumption of energy-dense snacks, drinks and fast foods [22]. A recent study on Saudi children aged 10-19 years has also reported a positively significant correlation between sugar-sweetened beverage consumption and poor dietary habits [6].

The formative years of adolescence represent a crucial stage in the human life cycle since it is the stage when lifestyles are formed and become established. During this period, adolescent becomes more independent and have increased access to food choices apart from those available at home. It is also in this period that adolescents increase their social interactions with peers of similar age and develop individual eating habits and physical-activity patterns. Dietary habits appear to be established in the mid-teens and were shown to be closely associated with lifestyle [23]. A better understanding of the relationships between healthy behaviors among young people is considered necessary for effective prevention and management of lifestyle-related risk factors. Some reports have even suggested that diet, physical activity and sedentary behaviors should be considered simultaneously in the prevention of cardiometabolic disease [20].

Saudi Arabia has witnessed tremendous lifestyle changes over the past few decades, and sedentary lifestyles are becoming particularly prevalent among Saudi children and youth. Data from a limited number of studies indicate that 60% of Saudi children and 71% of young people do not engage in physical activity of sufficient duration and frequency [24,25]. In addition, food-consumption patterns have changed dramatically in the Eastern Mediterranean countries during the past four decades, and calorie-dense foods and sugar-sweetened beverages are becoming increasingly accessible to children and adolescents [26]. Although there are some studies that examined certain aspects of the dietary and lifestyle habits among Saudi children and adolescents [6,10,27-30], the vast majority of these studies either had a small or unrepresentative sample size, used un-validated physical activity instruments or did not assess physical activity, sedentary behaviors and dietary habits simultaneously. Therefore, the purpose of the present study was to report on the prevalence of physical activity, sedentary behaviors and dietary habits among Saudi adolescents aged 14-19 years, using representative samples drawn from three major cities in Saudi Arabia. It was also the intent of this study to examine the interrelationships among the above-mentioned lifestyle factors.

## Methods

### Study sample

The present study is part of the Arab Teens Lifestyle Study (ATLS). ATLS is a school-based cross-sectional multicenter collaborative study [31]. The sample came from adolescent males and females enrolled in secondary schools from three major cities in Saudi Arabia: Riyadh is the capital of Saudi Arabia and the largest and most populated city in the country; Jeddah is the second-largest city and is located on the Red Sea in western Saudi Arabia; Al-Khobar is a modern city in the eastern province of Saudi Arabia. The minimum required sample size in each city was determined so that the sample proportion would be within ± 0.05 of the population proportion, with a 95% confidence level. For example, in the city of Riyadh, where the population of male students in public and private secondary schools was about 75,000, the necessary total sample size for male students was 382. Depending on the participating center, 10-15% additional students were included to compensate for missing data.

A multistage stratified random-sampling technique was used to select the sample. At the first stage, a systematic random-sampling procedure was used to select the schools. The schools were stratified into boys' and girls' secondary schools, with further stratification into public and private schools. The selection of the private and public schools was proportional to the population size. Four schools (two each from the boys' and girls' schools) were selected from each of the four geographic areas (east, north, south and west). At the second stage, classes were selected at each grade (level) using a simple random-sampling design. In this way, one class was randomly selected in each of the three grades (grades 10, 11 and 12) in each secondary school. Thus, we had a total selection of at least 24 classes in each city (12 each from the boys' and girls' schools). All students in the selected classes, who were free of any physical health problems, were invited to participate in the study. Owing to the differences in class size from city to city and from private to public schools, the sample sizes for the participating cities varied. In addition, some cities have more geographically dispersed schools, which required selecting more schools for better representation. The data were collected during October and November 2009. The study protocol was approved by the Research Center at King Saud University as well as by the General Directorate of School Education in the respective cities. In addition, all the schools and students consented to involvement in the survey. The total sample size consisted of 2908 adolescents, comprising 1401 males and 1507 females.

### Anthropometric measurements

Anthropometric variables included body weight and height. Measurements were performed in the morning by a trained researcher according to written standardized procedures. Body weight was measured to the nearest 100 g using calibrated portable scales. Measurements were done with minimal clothing and without shoes. Height was measured to the nearest centimeter using a calibrated measuring rod while the subject was in a full standing position without shoes. Body mass index (BMI) was calculated as the ratio of weight in kilograms by the height squared in meters. The International Obesity Task Force (IOTF) age- and sex-specific BMI cutoff reference standards [32] were used to identify overweight and obese adolescents between the ages of 14 and 17 years. For participants 18 years and older, we used WHO adult cutoff points of 25-29.9 kg/m^2 ^to define overweight and 30 kg/m^2 ^and higher for obesity [33].

### ATLS research instrument

The ATLS research instruments used for the collection of lifestyle information consisted of 47 items, including the first five items that the researcher had to measure and record. These items included age, weight, height, waist circumference and the student's level of study. Items 6 to 34 dealt with physical activity. Items 35 to 37 were questions on sedentary activity. Last, items 38 to 47 were specific questions on dietary habits. To ensure accurate and consistent measurements throughout this multicenter project, a standardized measurement protocol was employed in all participating data-collection centers. Written instructions were provided to the researchers on sampling procedures, weight, height and waist circumference measurements, and how to conduct the questionnaire. In addition, the participating centers were asked to avoid any data collection on months of hot, humid or very cold weather, since these environmental conditions may have a negative effect on the level of physical activity. Days on which midterm examinations were held were also avoided for the same reason.

### Physical-activity assessment

Due to the nature and diversity of the ATLS project, a self-reported questionnaire was used as the means to assess the level of physical activity of the young participants. The original questionnaire was previously shown to have a high reliability (ICC = 0.85; 95% CL = 0.70-0.93) and acceptable validity (r = 0.30; *p *< 0.05) against pedometer measurements using a convenient sample of young male 15- to 25-year-olds [34,35]. In another validity study, using both females and males aged 14-19 years, the current ATLS physical-activity questionnaire was also validated against electronic pedometer measurements [36] and was shown to have an acceptable validity coefficient (r = 0.37, *p *< 0.001).

The participants completed the questionnaire in their classrooms under the supervision of their teachers and in front of at least one of the research assistants. The questionnaire was designed to collect information on frequency, duration and intensity of a variety of light-, moderate- and vigorous-intensity physical activities during a typical week. The physical-activity questionnaire covered such domains as transport and household, fitness and sports activities. Physical activities were assigned metabolic equivalent (MET) values based on the compendium of physical activity [37] and the compendium of physical activity for youth [38]. Moderate-intensity physical activity includes activities such as normal-pace walking, brisk walking, recreational swimming, household activities and moderate-intensity recreational sports, such as volleyball, badminton and table tennis. Household activities were given a mean MET value of 3. This is because they include some items that may require less than 3 METs, such as washing dishes (2.5 METs), cleaning the bathroom (2.5 METs), cooking (2.5 METs) and ironing (2.3 METS) as well as other household activities that require 3 METs or more, such as car washing (3 METs), vacuuming (3.5 METs), mopping (3.5 METs) and gardening (3.5 METs). Moderate-intensity recreational sports were assigned an average MET value equivalent of 4 METs. Slow walking, normal-pace walking and brisk walking were assigned MET values of 2.8, 3.5 and 4.5 METs, respectively, based on modified METs values from the compendium of physical activity for youth [38]. Vigorous-intensity physical activity and sports included such activities as stair-climbing, jogging, running, cycling, self-defense, weight training and vigorous sports, such as soccer, basketball, handball and singles tennis. Vigorous-intensity sports were assigned an average MET value equivalent of 8 METs.

To measure the participants' levels of physical activity, we used the total METs-min per week and the METs-min per week spent in the moderate- and vigorous-intensity physical activities. To calculate the percentage of adolescents who met the daily physical-activity recommendations [13-16], we used two cutoff scores equivalent to 1 hour per day of moderate-intensity (4 METs) physical activity and 1 hour per day of moderate- to vigorous- intensity (6 METs) physical activity. We then converted these two amounts of exercise into METs-min per week, thus, corresponding to 1680 METs-min/week (60 min per day × 7 days per week × 4 METs) and 2520 METs-min per week (60 min per day × 7 days per week × 6 METs).

### Sedentary behaviors

Questions on sedentary behaviors followed the physical-activity questions and aimed to determine important information from adolescents related to the typical daily time spent on sedentary activities, including time spent viewing TV, playing video games, and computer and Internet use. Participants were asked to provide the average number of daily hours without differentiating between weekdays and weekends. For the total screen time cutoff points, we used the American Academy of Pediatrics guidelines of a maximum of 2 hours per day [39].

### Eating habits questionnaire

In addition to the physical-activity questionnaire, the ATLS questionnaire in a separate section included 10 specific questions designed to determine the frequency of certain dietary habits of adolescents. The questions included those related to how many times per typical week the participants consumed breakfast, sugar-sweetened drinks including soft beverages, vegetables (cooked and uncooked), fruit, milk and dairy products, donuts and cakes, candy and chocolate, energy drinks and fast foods. The fast foods in this regard included examples from both Western fast foods and Arabic fast-food choices, such as shawarma (grilled meat or chicken in pita bread with some salad). These questions covered healthy and unhealthy dietary habits. The students had a choice of answers, ranging from zero intake to a maximum intake of 7 days per week (every day). For the dietary cutoff points, we calculated the proportions of adolescents who had a daily intake of breakfast, fruit, vegetables and milk and those who exceeded 3 days' intake per week of the unhealthy dietary habits.

### Data and statistical analysis

Data at each center were checked and entered into a computer using standardized entry codes written on an SPSS (SPSS, Inc, Chicago, IL) data file. The entered data were then sent to one central processing location (Riyadh). At the central processing center, all the data were checked again for outliers and incorrect entries. To avoid over-reporting, physical-activity scores were cleaned and truncated at reasonable and realistic levels, taking into account the fairly long time spent in learning at schools, doing homework, sleeping and TV time. The maximum total time spent on physical activity per week was truncated at 1680 minutes (28 hours) or 4 hours of physical activity per day. In addition, the maximum number of stair levels taken by students per day was capped to 30 floors. The combined time of TV viewing, computer use and Internet time was also truncated at 16 hours per day.

Data were then analyzed using SPSS, version 15. Descriptive statistics were presented as means, standard deviations and proportions. Data that were not normally distributed, such as physical-activity scores in METs-min per week, were log-transformed before performing parametric analysis. Differences in anthropometric measurements between regions were tested separately for each of the males and females using one-way ANOVA. The proportion of males and females who exceeded specific cutoff scores in sedentary behaviors, physical activity and dietary habits was calculated. We used the *t *test for the two proportions to test the differences in the percentages of male and female adolescents who exceeded certain cutoff values for physical activity, screen time and dietary habits. In addition, a two-way ANOVA (two by three) with the Bonferroni test was used to test the differences in lifestyle factors relative to age and gender. We also used a two-way ANCOVA (two by three) while controlling for the effects of age to test the differences in lifestyle variables across gender (males and females) and cities (Al-Khobar, Jeddah and Riyadh). Finally, Pearson correlation coefficients were calculated for males and female separately to evaluate the associations among BMI and selected lifestyle factors. The level of significance was set at a *p *value of 0.05 or less.

## Results

Table 1 presents the anthropometric characteristics of the participants stratified by city and gender. Males from Riyadh but not females were slightly younger than those from Al-Khobar and Jeddah. Overall, males were taller, heavier and had significantly higher mean BMI value than females. The proportion of adolescents exceeding the specific cutoff values for the lifestyle factors is shown in Table 2. In all, 84% of males and over 91% of females watched TV and used the computer for more than 2 hours per day. That means only 16% of the males and less than 11% of females met the recommended screen time guidelines of 2 hours or less per day. Regarding daily physical-activity guidelines, about half of the males and less than a quarter of the females met the recommended 1 hour of moderate-intensity physical activity. When a level of daily moderate- to vigorous-intensity physical activity was considered as a cutoff value, even lower proportions of males (43.5%) and females (12.9%) were able to meet the physical-activity guidelines.

**Table 1:** Anthropometric characteristics of the participants.

City	Gender	N	Age (yr)	Weight (kg)	Height (cm)	BMI (kg/m^2^)
Al-Khobar	Male	348	16.8 ± 1.1 ^a^	69.9 ± 20.8	168.7 ± 7.9	24.5 ± 6.8
	Female	367	16.6 ± 1.0	57.2 ± 13.9 ^b^	157.7 ± 6.1 ^b^	23.0 ± 5.4 ^b^
Jeddah	Male	560	16.8 ± 1.0 ^a^	71.4 ± 20.7	168.4 ± 6.6	25.1 ± 6.8
	Female	632	16.5 ± 1.1	57.4 ± 15.7 ^b^	155.9 ± 5.8 ^b^	23.6 ± 6.3 ^b^
Riyadh	Male	493	16.6 ± 1.2 ^a^	68.3 ± 19.9	168.1 ± 7.3	24.1 ± 6.6
	Female	508	16.5 ± 1.0	59.2 ± 16.1 ^b^	156.8 ± 5.8 ^b^	24.0 ± 6.2 ^b^

All	Male	1401	16.7 ± 1.1 ^c^	70.0 ± 20.5 ^c^	168.4 ± 7.2 ^c^	24.6 ± 6.7 ^c^
	Female	1507	16.5 ± 1.1	57.9 ± 15.5	156.7 ± 5.9	23.6 ± 6.1

Data are means and standard deviations (total n = 2908).

BMI = body mass index.

One-way ANOVA tests: ^a ^and ^b ^= significant difference between cities at *p *< 0.05 for males and females, respectively.

*t *tests for independent samples: ^c ^= significant gender difference between males and females at *p *< 0.05.

**Table 2:** The proportions (%) of Saudi adolescents who exceeded certain cut-off values for physical activity, screen time and dietary habits.

Variable	Males	Female
> 2 hours of screen time	84.0	91.2 **
> 1680 METs-min/week**^1^**	55.5	21.9 **
> 2520 METs-min/week**^2^**	43.5	12.9 **
Daily breakfast intake	28.7	20.6 **
Daily vegetables intake	23.3	22.3
Daily fruits intake	16.0	9.6 **
Daily milk Intake	33.2	25.1 **
Sugar-sweetened drinks intake (> 3 day/week)	67.3	57.4 **
Fast food intake (> 3 day/week)	30.2	24.9 **
French fries/potato chips intake (> 3 day/week)	25.0	30.7 **
Cake/donut/biscuit intake (> 3 day/week)	24.8	28.8 *
Sweets/chocolates intake (> 3 day/week)	37.3	52.6 **
Energy drinks intake (> 3 day/week)	16.3	8.5 **

**^1 ^**= 60 min per day × 7 days/week × 4 METs (moderate-intensity physical activity).

**^2 ^**= 60 min per day × 7 days/week × 6 METs (moderate- to vigorous-intensity physical activity),

* *p *< 0.05

** *p *< 0.01

There were significantly more males than females who met the cutoff scores for most of the dietary habits, including intake of daily breakfast, fruit, vegetables, milk and dairy products, sugar-sweetened drinks, fast foods and energy drinks. However, significantly more females than males exceeded the cutoff scores of no more than 3 days' intake per week of the rest of the dietary habits.

It is worth noting that two-thirds (67.3%) of males and about 60% of females consumed sugar-sweetened drinks more than 3 days per week. The results (not shown in Table 2) also revealed that 41% of males and 31% of females consumed sugar-sweetened drinks on a daily basis. Furthermore, more than 30% of males and about 25% of females consumed fast foods more than three times per week. The proportions of males and females who ate fast foods on a daily basis were 9% and 6%, respectively. From the consumption frequencies of candy, chocolate and energy drinks shown in Table 2, there are two contrasting findings. The intake of candy and chocolate among females was significantly (*p *< 0.01) higher than among males, while many more males than females consumed energy drinks.

Table 3 shows the mean values for the sedentary behaviors, physical-activity indices and dietary habits grouped by city and gender. There were significant gender and location interaction effects for most of the studied lifestyle variables. This means that the males and females were responding differently to the lifestyle factors within the three cities. Additionally, the main effects of age appeared significant with most of the physical-activity measures and dietary habits. For this reason, we controlled for the effect of age when comparing the lifestyle factors across the genders and cities. Compared with males, females on average were significantly (*p *< 0.05) more sedentary (6.6 versus 5.3 hours/week, for the combined TV time and computer use), much less active (1211.1 versus 3051.4 METs-min/week), especially with vigorous-intensity physical activities (554.4 versus 2149.9 METs-min/week) and on fewer days per week consumed breakfast (3.71 versus 3.29), fruit (3.27 versus 2.52), milk and diary products (4.31 versus 3.66), sugar-sweetened drinks (4.75 versus 4.25), fast foods (2.90 versus 2.63) and energy drinks (1.55 versus 0.89). However, the females' weekly intake of French fries and potato chips, cakes and donuts, and candy and chocolate was significantly (*p *< 0.05) higher than the males'. The total sedentary time for the females was about 124% that of the males, while the total energy expenditure in METs-min per week for the females was about 40% that of the males. In terms of minutes per week (not shown in Table 3), the total physical-activity time for the males and females was 503.3 ± 475.4 and 265.9 ± 313.0 minutes, respectively, or about 72 and 38 minutes of daily physical activity for the males and females, respectively.

**Table 3:** Sedentary behaviors, physical activity measures and dietary habits for Saudi adolescents grouped by city and gender.

Variable	Al-Khobar		Jeddah		Riyadh		All	
	
	Male	Female	Male	Female	Male	Female	Male	Female
TV Viewing (hours/day)*^**b, c, d**^*	2.77 ± 2.2	3.64 ± 2.4	2.60 ± 1.9	3.10 ± 2.2	2.69 ± 1.9	2.75 ± 2.0	2.67 ± 2.0	3.11 ± 2.2
Computer use (hours/day)^***b, d***^	2.67 ± 2.4	3.27 ± 2.7	2.76 ± 2.5	3.43 ± 2.7	2.55 ± 2.1	3.70 ± 2.5	2.67 ± 2.3	3.49 ± 2.7
METs-min/week of Moderate -intensity physical activity^***b, c, d***^	1199.4 ± 1230.7	735.3 ± 878.5	1020.4 ± 1028.5	799.8 ± 869.6	847.0 ± 1021.9	698.5 ± 882.7	1003.4 ± 1087.6	751.9 ± 876.4
METs-min/week of Vigorous -intensity physical activity *^**a, b, c, d**^*	2271.9 ± 2407.3	406.1 ± 726.4	2331.4 ± 2416.4	625.9 ± 1032.5	1856.1 ± 2167.1	568.2 ± 742.2	2149.9 ± 2338.1	554.4 ± 878.7
Total METs-min/week ^***a, b, c, d***^	3363.2 ± 3016.3	984.0 ± 1298.3	3244.3 ± 2921.8	1370.9 ± 1565.9	2613.2 ± 2741.2	1171.4 ± 1303.0	3051.4 ± 2921.9	1211.1 ± 1426.8
Breakfast consumption (frequency/week) ^***a, b, c, d***^	3.79 ± 2.7	2.95 ± 2.6	3.06 ± 2,6	3.04 ± 2.5	4.42 ± 2.6	3.84 ± 2.5	3.71 ± 2.7	3.29 ± 2.6
Vegetables Consumption (frequency/week) ^***a***^	3.58 ± 2.4	3.40 ± 2.4	3.76 ± 2.4	3.59 ± 2.4	3.74 ± 2.4	3.85 ± 2.3	3.71 ± 2.4	3.63 ± 2.4
Fruits Consumption (frequency/week) ***^a, b^***	3.35 ± 2.3	2.45 ± 2.1	3.29 ± 2.2	2.61 ± 2.1	3.19 ± 2.3	2.45 ± 2.1	3.27 ± 2.3	2.52 ± 2.1
Milk/dairy products intake (frequency/week) *^**a, b, c, d**^*	4.53 ± 2.4	3.33 ± 2.6	4.10 ± 2.5	3.48 ± 2.5	4.40 ± 2.5	4.10 ± 2.5	4.31 ± 2.4	3.66 ± 2.5
Sugar-sweetened drinks (frequency/week) *^**a, b, c**^*	4.82 ± 2.3	4.46 ± 2.3	4.45 ± 2.5	3.97 ± 2.3	5.03 ± 2.2	4.45 ± 2.3	4.75 ± 2.4	4.25 ± 2.3
Fast foods (frequency/week) *^**b**^*	2.77 ± 1.9	2.70 ± 1.9	2.97 ± 2.0	2.65 ± 1.9	2.90 ± 2.0	2.54 ± 1.8	2.90 ± 2.0	2.63 ± 1.8
French fries/potato chips (frequency/week) ^***b, d***^	2.36 ± 2.0	3.04 ± 2.2	2.71 ± 2.1	2.73 ± 2.0	2.31 ± 2.0	2.84 ± 2.0	2.48 ± 2.1	2.84 ± 2.0
Cake/donuts (frequency/week)*^b^*	2.52 ± 2.2	2.89 ± 2.1	2.42 ± 2.1	2.71 ± 2.0	2.46 ± 2.0	2.68 ± 2.0	2.46 ± 2.1	2.74 ± 2.1
Sweets (frequency/week) *^**b, d**^*	3.08 ± 2.3	3.67 ± 2.3	3.15 ± 2.4	3.77 ± 2.3	2.89 ± 2.3	4.35 ± 2.2	3.04 ± 2.3	3.95 ± 2.3
Energy drinks (frequency/week)***^b, d^***	1.42 ± 2.2	1.08 ± 1.9	1.71 ± 2.2	0.85 ± 1.6	1.47 ± 2.1	0.81 ± 1.6	1.55 ± 2.2	0.89 ± 1.7

Data are means and standard deviations

Two-way ANCOVA tests controlling for the effect of age: ***a ***= significant differences at *p *< 0.05 for the effect of age; ***b ***= significant differences at *p *< 0.05 for the main effect of gender; ***c ***= significant differences at *p *< 0.05 for the main effect of city; ***d ***= significant differences at *p *< 0.05 for the effect of the interaction (gender by city).

The sedentary behaviors, physical activity measures and dietary habits relative to age and gender are shown in Table 4. Two-way ANOVA analysis revealed that only computer use and intake of breakfast and fast foods exhibited significant age and gender interactions. No significant differences relative to age or gender were seen in TV viewing and intake of sugar-sweetened drinks, French fries and potato chips, and cakes and donuts. Furthermore, except at age 14 years, there is a tendency for the total physical-activity levels and the intake of breakfast and fruit to decline, while the consumption of sugar-sweetened drinks tends to increase with increasing age in males. The consumption of fruit, on the other hand, appears to decrease with increasing age among females. There were obviously large differences between males and females in terms of total and vigorous physical activity, and this amounted to a threefold difference in some age-groups. In addition, the frequency of females' intake of energy drinks was almost half that of males' across all ages. Overall, the effect of age was significantly (*p *< 0.05) present in all the physical-activity indices and half of the dietary habits.

**Table 4:** Sedentary behaviors, physical activity measures and dietary habits for Saudi adolescents grouped by age and gender.

Variable	Gender	Age in years					
		
		14	15	16	17	18	19
TV Viewing time (hours/day)	M	2.00 ± 2.5	2.66 ± 2.0	2.64 ± 1.9	2.73 ± 1.9	2.54 ± 2.0	3.1 ± 2.1
	
	F	2.06 ± 1.6	3.19 ± 2.1	3.2 ± 2.3	2.97 ± 2.1	3.10 ± 2.3	3.39 ± 2.4

Computer use (hours/day)^***b, c***^	M	3.00 ± 1.6	2.51 ± 2.3	2.61 ± 2.3	2.65 ± 2.2	2.94 ± 2.6	2.38 ± 2.5
	
	F	4.59 ± 3.1	3.35 ± 2.4	3.62 ± 2.8	3.56 ± 2.7	3.06 ± 2.4	3.51 ± 2.4

METs-min/week of Moderate -intensity physical activity *^**a**^*	M	815.1 ± 553.9	1104.5 ± 1135.6	1018.5 ± 1110.5	919.6 ± 923.8	1085.5 ± 1266.6	784.1 ± 890.5
	
	F	778.3 ± 1089.2	818.1 ± 1020.2	791.8 ± 897.0	647.2 ± 754.1	817.9 ± 914.1	832.1 ± 831.7

METs-min/week of Vigorous -intensity physical activity *^**a, b**^*	M	1793.1 ± 1222.3	2308.6 ± 2264.9	2292.3 ± 2465.0	2101.0 ± 2383.5	2008.8 ± 2141.5	1635.5 ± 2116.6
	
	F	359.9 ± 360.0	582.5 ± 916.3	636.5 ± 1012.8	479.8 ± 736.6	562.5 ± 865.9	274.3 ± 310.4

Total METs-min/week ^***a, b***^	M	2608.2 ± 1161.5	3295.5 ± 2850.8	3237.5 ± 3046.4	2907.3 ± 2851.5	2995.6 ± 2912.7	2258.9 ± 2662.9
	
	F	1094.9 ± 1239.0	1272.9 ± 1638.0	1348.8 ± 1560.9	1035.8 ± 1166.6	1264.7 ± 1466.8	1002.4 ± 874.0

Breakfast consumption (frequency/week)***^a, b, c^***	M	4.5 ± 2.8	4.1 ± 2.7	4.1 ± 2.7	3.3 ± 2.6	3.7 ± 2.7	3.5 ± 2.8
	
	F	3.9 ± 2.6	3.6 ± 2.5	3.2 ± 2.6	3.4 ± 2.6	3.2 ± 2.6	1.8 ± 2.2

Vegetables Consumption (frequency/week) ^***a***^	M	3.8 ± 2.2	4.1 ± 2.5	3.7 ± 2.4	3.6 ± 2.4	3.7 ± 2.3	3.1 ± 2.3
	
	F	3.5 ± 2.1	3.9 ± 2.4	3.6 ± 2.3	3.5 ± 2.3	3.7 ± 2.4	2.9 ± 2.5

Fruits Consumption (frequency/week) ***^a, b^***	M	4.2 ± 2.4	3.7 ± 2.4	3.3 ± 2.2	3.2 ± 2.3	3.2 ± 2.3	2.6 ± 2.2
	
	F	2.9 ± 2.1	2.7 ± 2.3	2.6 ± 2.1	2.4 ± 2.1	2.4 ± 2.1	2.2 ± 2.3

Milk/dairy products intake (frequency/week) *^**a, b**^*	M	4.8 ± 2.2	4.4 ± 2.5	4.2 ± 2.4	4.3 ± 2.4	4.6 ± 2.4	3.6 ± 2.7
	
	F	4.2 ± 2.4	4.0 ± 2.4	3.6 ± 2.5	3.7 ± 2.5	3.5 ± 2.7	2.2 ± 2.4

Sugar-sweetened drinks (frequency/week)	M	3.5 ± 2.4	4.4 ± 2.3	4.7 ± 2.3	4.9 ± 2.4	4.8 ± 2.4	4.9 ± 2.4
	
	F	4.6 ± 2.6	4.0 ± 2.4	4.3 ± 2.3	4.2 ± 2.3	4.2 ± 2.4	4.7 ± 2.1

Fast foods (frequency/week) ***^a, c^***	M	2.3 ± 1.0	2.4 ± 1.9	2.9 ± 1.9	3.1 ± 2.0	3.0 ± 1.9	2.6 ± 2.0
	
	F	3.3 ± 2.0	2.5 ± 1.7	2.7 ± 1.9	2.6 ± 1.9	2.4 ± 1.6	2.9 ± 2.0

French fries/potato chips (frequency/week)	M	2.7 ± 2.4	2.3 ± 2.0	2.5 ± 2.1	2.6 ± 2.1	2.5 ± 2.1	2.5 ± 2.1
	
	F	2.7 ± 2.2	2.7 ± 2.0	2.9 ± 2.0	2.9 ± 2.0	2.8 ± 2.1	3.0 ± 2.0

Cake/donuts (frequency/week)	M	2.5 ± 1.9	2.1 ± 1.8	2.4 ± 2.0	2.6 ± 2.1	2.6 ± 2.2	2.5 ± 2.1
	
	F	2.7 ± 1.7	2.6 ± 2.1	2.7 ± 2.1	2.9 ± 2.1	2.7 ± 1.9	2.5 ± 2.1

Sweets (frequency/week) *^**a, b**^*	M	2.3 ± 1.3	2.9 ± 2.3	3.2 ± 2.3	3.1 ± 2.3	3.0 ± 2.3	2.2 ± 2.3
	
	F	3.4 ± 2.1	3.9 ± 2.1	4.1 ± 2.3	4.0 ± 2.3	3.8 ± 2.3	3.7 ± 2.5

Energy drinks (frequency/week)***^b^***	M	1.8 ± 2.3	1.3 ± 2.1	1.5 ± 2.0	1.7 ± 2.3	1.6 ± 2.1	1.8 ± 2.2
	
	F	1.1 ± 1.8	0.70 ± 1.5	0.99 ± 1.9	0.78 ± 1.6	1.0 ± 1.8	1.2 ± 1.8

Data are means and standard deviations.

Two-way ANOVA tests: ***a ***= significant differences at *p *< 0.05 for the main effect of age; ***b ***= significant differences at *p *< 0.05 for the main effect of gender; ***c ***= significant differences at *p *< 0.05 for the effect of the interaction (age by gender).

Table 5 presents the results of correlation coefficient analysis of BMI and selected lifestyle variables separately for males and females. BMI shows a significant inverse correlation with the intake of breakfast, milk and diary products, candy and chocolate, and physical activity in males and with the intake of breakfast, fast foods, sugar-sweetened drinks, French fries and potato chips, cakes and donuts, and candy and chocolate in females. Screen time (the combined time of TV viewing, playing video games and computer use) exhibited a significant positive relationship with the intake of fast foods, sugar-sweetened drinks, French fries and potato chips, cakes and donuts, candy and chocolate, and energy drinks among both males and females. The strength of the correlation was higher in females than in males. Furthermore, among females, screen time showed a significant negative correlation with the intake of breakfast, vegetables, fruit, and milk and diary products. No significant relationship was found between screen time and physical activity in either the males or females. Both the total physical activity and vigorous physical activity had a significantly positive correlation with the intake of unhealthy dietary items, except sugar-sweetened drinks in males and with the intake of fruit, vegetables and energy drinks in females. Breakfast intake showed a positive correlation with almost all other dietary habits, except fast foods, French fries and potato chips, and candy and chocolate in males and French fries and potato chips, cakes and donuts, and candy and chocolate in females. Moreover, fast foods exhibited a high positive correlation with French fries and potato chips, cakes and donuts, sugar-sweetened drinks, candy and chocolate, and energy drinks, while physical activity showed a positive correlation with the intake of fruit and vegetables in both genders. This perhaps suggests clustering effects for the healthy versus unhealthy lifestyle habits.

**Table 5:** Correlation coefficients of BMI and selected lifestyle variables separately among male and female adolescents.

Variables	Body mass Index	Screen time (TV + computer)	Breakfast	Vegetables	Fruits	Milk & dairy products	Fast foods	Sugar-sweetened beverages	French fries & potato chips	Cake & donuts	Sweets	Energy drinks	Total METs-min/week	METs-min/week vigorous activity
**Body mass index**	**1**	**-0.03**	-0.09***	**-0.01**	**0.01**	**-0.01**	-0.08**	-0.10***	-0.13***	-0.11***	-0.17***	**-0.01**	**0.02**	**-0.01**

**Screen time (TV+PC use)**	0.02	**1**	-0.08**	-0.12***	-0.07**	-0.09***	0.31***	0.19***	0.22***	0.14***	0.23***	0.21***	**-0.04**	**-0.02**

**Breakfast**	-0.13***	-0.02	**1**	0.27***	0.24***	0.38***	0.08**	-0.14***	**-0.03**	**0.02**	**0.02**	-0.15***	**0.03**	**0.02**

**Vegetables**	-0.03	0.01	0.20***	**1**	0.47***	0.34***	-0.12***	**-0.04**	**-0.04**	0.05*	**0.00**	-0.12***	0.14***	0.11***

**Fruits**	-0.02	0.01	0.19***	0.54**	**1**	0.28***	-0.05*	-0.09***	**-0.02**	0.10***	**0.00**	-0.06*	0.19***	0.16***

**Milk & dairy products**	-0.07**	-0.02	0.29***	0.40***	0.35***	**1**	-0.10***	**-0.03**	-0.05*	0.09***	**0.03**	-0.15**	0.08**	**0.03**

**Fast foods**	-0.03	0.22***	-0.03	0.01	0.06*	0.04	**1**	0.37***	0.52***	0.30***	0.30***	0.34***	**0.01**	**0.02**

**Sugar-sweetened beverages**	-0.03	0.17***	0.09**	0.06*	0.02	0.09**	0.42***	**1**	0.36***	0.23***	0.32***	0.23***	**-0.04**	**-0.04**

**French fries & potato chips**	-0.02	0.15***	-0.00	0.12***	0.13***	0.14***	0.47***	0.31***	**1**	0.37***	0.38***	0.28***	**0.03**	**0.03**

**Cake & donuts**	-0.04	0.17***	0.08**	0.13***	0.16***	0.17***	0.30***	0.27***	0.40***	**1**	0.53***	0.16***	**0.01**	**0.00**

**Sweets**	-0.07**	0.18***	0.03	0.08**	0.16***	0.16***	0.35***	0.31***	0.37***	0.52***	**1**	0.15***	**0.02**	**0.02**

**Energy drinks**	0.01	0.23***	-0.10***	-0.03	0.07**	-0.07**	0.34***	0.25***	0.27***	0.21***	0.26***	**1**	0.06*	0.07*

**Total METs-min/week**	-0.10***	-0.01	0.11***	0.17***	0.23***	0.19***	0.09**	0.04	0.09**	0.07*	0.08**	0.09**	**1**	0.83***

**METs-min/week vigorous activity**	-0.14***	-0.01	0.11***	0.15***	0.20***	0.16***	0.08**	0.04	0.09**	0.05*	0.07**	0.06*	0.95***	**1**

Females' results are shown on the upper right side with bold fonts.

* *p *< 0.05; ** *p *< 0.01; *** *p *< 0.001

## Discussion

Despite the enormous lifestyle changes experienced by Saudi society during recent decades, few researches have simultaneously been conducted on the physical activity, sedentary behaviors and dietary habits of Saudi adolescents. The present study reported on the prevalence of the above lifestyle factors among adolescents aged 14-19 years from three major cities in Saudi Arabia. The findings of this study provide evidence on the high prevalence of sedentary behaviors and the low level of physical activity, especially among females. Unhealthy dietary habits were also widely found among both genders. Furthermore, correlation analyses revealed that unhealthy behaviors, such as increased screen time and unhealthy dietary habits, appear to aggregate in this group of Saudi adolescents.

### Physical activity

Although some health benefits can occur through an average of 30 minutes of physical activity per day [14], physical-activity guidelines for children and adolescents recommend that they should participate in at least 60 minutes of moderate to vigorous physical activity on a daily basis [13-16]. In the present study, we used 1680 METs-min per week as cutoff scores to correspond to 1 hour of daily moderate-intensity physical activity and 2520 METs-min per week as cutoff scores corresponding to 1 hour of daily moderate- to vigorous-intensity physical activity. Based on these cutoff scores, we found a considerably high prevalence of physical inactivity, especially among Saudi females. About half of the males and less than quarter of the females met the current recommendations of 1 hour daily of moderate-intensity physical activity. Such high rates of low physical activity levels represent an area of great concern because of the association of inactivity with increased cardiovascular and metabolic risk factors in children and adolescents [13,15]. Previous local data using objective physical-activity measurement indicated that 60% of Saudi children and 71% of youth do not engage in health-enhancing physical activity of sufficient duration and frequency [24,25]. Major factors that contribute to youth inactivity in Saudi Arabia include a reliance on cars rather than walking for short-distance travel, including trips to and from school [40], and limited quality physical education programs in schools, especially for girls.

It is well understood that a comparison between physical-activity studies should be made with caution since wide variations are observed in age range, representation and activity-assessment methods. In spite of that, it is important to provide comparative estimates of physical-activity levels in different countries to place our sample prevalence into perspective. Findings from the European Youth Heart Study using an accelerometer for physical-activity measurements showed that the great majority of 16-year-old boys (81.9%) and girls (62.0%) achieved current health-enhancing physical-activity recommendations [41]. In the United States, results from the Youth Risk Behavior Surveillance indicated that only 18.4% of adolescents met these physical-activity guidelines [42]. Furthermore, more than 52% of Greek-Cypriot children and adolescents met the physical-activity guidelines [43]. In Finland, among 15- to 16-year-olds, 59% of boys and 50% of girls reported 60 minutes or more of total physical activity per day; however, when daily moderate- to vigorous-intensity physical activity was considered, lower proportions of the boys (23%) and girls (10%) were able to meet the recommended amount of daily physical activity [44]. The Global School-based Student Health Survey (GSHS), which included data on physical activity and sedentary behaviors of schoolchildren in 34 countries, indicated that nearly 24% of boys and over 15% of girls across the countries met the physical-activity recommendations [45].

Females in the present study were found to be not just significantly more sedentary than males, but they were much less physically active too, especially with vigorous physical activity. Insufficient vigorous physical activity was shown to be a risk factor for higher BMI for adolescent boys and girls [46]. Thus, our findings suggest that Saudi females may be a good target for physical-activity intervention. It is noteworthy that physical-activity levels of Arab females, irrespective of the region, have generally been reported to be much lower than those of males [6,47,48]. This could be due to the fact that females have generally limited opportunities compared with males to engage in physical activity, both in and outside school. Not many female schools in Saudi Arabia offer physical education classes. In addition, for cultural reasons, families may not encourage females to take part in physical activity. Elsewhere, males were found more likely than females to participate in sports and physical activity [43,49].

Modern life has to a great extent systematically reduced total energy expenditure. Moreover, urbanization and related environmental determinants may also be considered an important risk factor for physical inactivity in developing countries undergoing economic transition [50]. Before the recent economic growth surge, which started three decades ago, communities in major cities in Saudi Arabia were designed to support pedestrian travel in common daily activities. These traditional design settings facilitate walking and cycling, resulting in an increase in daily living activity. Walking and cycling to and from school was also common in the past. In contrast, major Saudi Arabian cities are now modernized, with large street networks and separate zoning for residential and commercial areas. This kind of design requires the use of automobiles for all trips and, therefore, totally discourages walking.

### Sedentary behaviors

The prevalence of sedentary behaviors found in the present study among Saudi adolescents was remarkably high. The American Academy of Pediatrics (AAP) has expressed concern about the amount of time that children and adolescents spend viewing TV and has issued guidelines recommending that screen time not exceed 2 hours per day [39]. Only 16% of adolescent males and less than 11% of females in the present study actually met the AAP recommendations on daily screen time. The implication of this finding is that there is a need to reduce the time spent by adolescents on TV viewing and computer use. Excessive TV viewing in adolescence appears to be related to an unfavorable cardiovascular risk factor profile [51]. In addition, it is now recognized that sedentary behaviors are associated with harmful health outcomes that are different from those attributable to the lack of physical activity [17].

In comparison with our findings on the prevalence of screen time among adolescents, much less prevalence of TV viewing was reported for Saudi adolescents in the city of Abha, where 38% of the participants watched TV for more than 3 hours per day [30]. The Abha sample, however, included only boys with a wider age range (11-19 years) located in southwestern Saudi Arabia. In addition, watching TV was found to be the predominant leisure time pursuit among 14- to 16-year-old adolescents in the United Arab Emirates, with an average of 2.5 hours per day [48]. Elsewhere, more than half (52.4%) of Greek-Cypriot adolescents met the recommendations of the AAP on TV viewing [43]. Among adolescent Finns, 48% of boys and 44% of girls reported watching TV more than 2 hours per day [44]. The prevalence of 2 hours or more screen time per day for Chinese boys and girls aged 13-18 years was 44.3% and 34.7%, respectively [52]. The proportion of US youth who met the TV-viewing guideline of 2 hours or less per day ranged from 65% to 71% [53]. Among Canadian youth, 41% of girls and 34% of boys in grades 6-10 watched TV 2 hours or less per day; but when the total screen time was considered, only 18% of girls and 14% of boys met the 2-hours guideline [54]. The mean daily time spent on TV viewing by Italian youth was reported as 2.8 hours [55], which is very similar to what was found in the present study. However, much less time, with an average of 4.7 hours per week, was spent on all sedentary activities by Hungarian youth aged 13-19 years [56]. From the preceding comparison, it appears that the prevalence rate of screen time among Saudi adolescents exceeds that of adolescents in many countries and is comparable with the prevalence rate observed among adolescents in the United States and Canada.

The females in the present study spent, on average, more screen time than the males--a finding that is supported by some previous studies [43,54,56]. However, not all studies found such a gender difference [57]. Contrary to our findings, male high school students from Turkey had higher screen time than females [58]. Females in the present study were found to be much less active than males, and compared with males they had less opportunity to engage in physical activity; this would suggest that females may substitute physical activity with sedentary activity. However, examining the association between sedentary behaviors and physical activity in our sample did not reveal any significant relationship between the two variables, indicating that other independent factors may be responsible for this gender difference in screen time.

### Dietary habits

The majority (ranging from 66.85 to 80.4%) of adolescents in the current study did not daily consume breakfast, fruit, vegetables and milk, while a considerable proportion frequently showed unhealthy dietary habits. The WHO Global Strategy for Diet and Physical Activity recommendations call for achieving an energy balance, limiting the energy intake from fats, reducing the intake of free sugars and increasing fruit and vegetable consumption [3]. It is worth noting, though, that the food-consumption pattern has changed dramatically in Eastern Mediterranean countries during the past four decades. During the period from 1970 to 2005, the intake of animal products and refined sugar increased while the intake of fruit and vegetables and complex carbohydrates decreased [26].

Presently, unhealthy dietary habits are not uncommon in youth. The results from the 2009 National Youth Risk Behavior Surveillance of the United States indicated that during the 7 days preceding the survey, about 78% of high school students had not eaten fruit and vegetables five or more times per day and 29.2% had drunk soda or pop at least once per day [42]. In the present study, the prevalence of daily fruit and vegetable consumption by both genders was noticeably low, and it was slightly lower than what was previously reported for Saudi adolescents in Jeddah, which amounted to 27.6% and 26.4% for fruit and vegetables, respectively [59]. However, our sample prevalence is much lower than the 50.8% and 62.4% prevalence reported for fruit and vegetable intake by Saudi adolescents from Abha [30]. Moreover, fruit and vegetable consumption more than three times per week among adolescent males and females in the United Arab Emirates was reported to range from 49% to 69% [11]. This figure is higher than the average prevalence of fruit (32%) and vegetable (48%) consumption more than three times per week found in the present study. Skipping breakfast is another unhealthy dietary habit and was found to be very common in the present study. Breakfast skipping was reported to be 49% among Saudi adolescents from Abha [29], about 15% among Saudi secondary school students from Jeddah [10], and about 10% among adolescent males and nearly 19% among females in the United Arab Emirates [11]. Skipping breakfast was also shown to be prevalent in the United States and Europe, ranging from 10% to 30%, depending on age-group, population and definition [9].

The overall weekly frequency of fast-food intake in the present study was significantly higher in males than in females (2.90 versus 2.63). This may be due to the fact that for cultural reasons, adolescent males in Saudi Arabia have more opportunity than females to go outside to fast-food restaurants. Irrespective of the difference between males and females, such frequency is much lower than the average frequency of fast-food intake of 4.5 times per week recently reported for adolescents in Riyadh [6] and higher than the average frequency that was reported for Saudi adolescents in another study [29]. In addition, daily consumption of fast food by adolescents in the present study (9% for males and 6% for females) is not much different from that reported for Saudi secondary school boys in Riyadh [27], but it is much less than the 30% that was recently reported for adolescents in Abha [30]. The proportion of adolescents in the present study consuming fast food more than three times per week was 30.2% and 24.9% for males and females, respectively. These percentages are lower than the rate of fast-food intake reported recently for Emirates adolescents [11].

In recent years, high-energy food snacks have become readily available for the majority of Saudi children and adolescents. In the present study, among unhealthy dietary items consumed more than 3 days per week, the highest were sugar-sweetened beverages (67.2% and 57.4% among males and females, respectively) and candy and chocolate (37.3% and 52.6% among males and females, respectively). Previous local studies reported a proportion of daily consumption of soft drinks by Saudi adolescents ranging from as little as 33.4% [27] to as high as 62.4% [30]. Furthermore, the average soft drink and candy intake more than three times per week among adolescents in the United Arab Emirates was found to be 63.2% and 62.9%, respectively [11].

### Relationships amongst lifestyle factors

We observed a significant negative association between breakfast intake and BMI in the adolescent males and females. Previous reports indicated that breakfast intake was negatively related to overweight in adolescents [7,60]. However, the inverse relationship found in the present study between females' BMI and the intake of fast foods, sugar-sweetened beverages, French fries and potato chips, and cakes and donuts cannot be really explained. In an international comparative study involving youth from 34 countries, Janssen et al. [61] observed a significant negative relationship between BMI categories and candy consumption in 91% of countries, while no association between the consumption of non-diet soft drinks and being overweight was found.

In the present study, breakfast, fruit and vegetable consumption showed significantly positive correlations with physical activity in males and females and significantly inverse relationships with screen time in females. Ample evidence shows that high physical-activity levels are associated with healthy dietary habits. Among adolescents from Riyadh, Collison et al. [6] found that exercise was positively correlated with fruit, vegetable and cereal intake in both genders. In French adolescents, it was reported that physical activity positively correlated with the consumption of fruit and vegetables [20]. Adolescents from the United States participating in high school sports were also more likely than nonparticipants to report fruit and vegetable consumption the previous day [62]. In addition, Kremers et al. [63] showed that a low frequency of fruit consumption was associated with low physical activity among Dutch adolescents. In a study conducted on Iranian adolescents, it was shown that the most active adolescents consumed fruit, vegetables and dairy products more frequently than their less active peers. [64]. Fruit, vegetable and milk intake was shown to inversely relate to fast-food intake among Saudi adolescents in Riyadh [6]. It is worth noting, however, that the most consistent determinants of fruit and vegetable consumption in children and adolescents were identified as age (younger children), gender (being a girl), socioeconomic status, preference, parental intake and availability [65].

The present study did not find any significant association between sedentary behaviors as measured by total screen time and total or vigorous physical activity (r value ranged from -0.01 to 0.04). Research on the relationships between sedentary behaviors and physical activity has produced contrasting results. Some studies have shown an association [44,66], but other studies have failed to show any relationship between the two variables [21,57]. Findings from a study on French pre-adolescents indicated that sedentary behaviors and physical activity are considered distinct behaviors [20]. According to Gordon-Larson et al. [49], physical activity and sedentary activity were associated with very different determinants: physical activity was associated with environmental factors while inactivity was most associated with sociodemographic factors. The implication of the absence of a relationship between screen time and physical activity found in the present study is that both physical inactivity and sedentary behaviors must be targeted when implementing any program for the purpose of promoting physical activity and reducing sedentary behaviors.

The findings that screen time and fast-food intake in the present study both exhibited a significantly positive relationship with consumption of French fries and potato chips, cakes and donuts, sugar-sweetened beverages, candy and chocolate, and energy drinks indicates clustering effects of unhealthy dietary habits. Platat et al. [20] reported a significant positive association with the intake of French fries and potato chips and sweetened drinks and a negative association with the high consumption of fruit and vegetables. In our study, screen time was also inversely related to the intake of fruit and vegetables (*p *> 0.01). The results of a systematic review indicated that screen time in adolescents was associated with unhealthy dietary habits, including lower fruit and vegetable intake and higher consumption of energy-dense snacks, drinks and fast foods [22]. Furthermore, a recent study on Saudi children aged 10-19 years has also reported a positively significant correlation between sugar-sweetened beverage consumption and poor dietary habits [6].

### Strength and limitations

The findings of the present study should be seen in the light of their strengths and limitations. This is a novel study in Saudi Arabia that collectively examined several lifestyle factors in a large and representative sample of Saudi adolescents, using a validated and comprehensive physical-activity questionnaire, employing metabolic equivalents for calculating energy expenditure from physical activity. The information stemming from this study should add to existing knowledge about the lifestyle factors in a society experiencing a nutrition transition. One of the limitations of this multicenter epidemiological study was that the information was based on self-report, although we made every effort to minimize any possible over- or under-reporting by the participants. Because this is a cross-sectional study, the temporality of the associations between sedentary behaviors, physical activity and dietary habits cannot be certain; however, many of the observed associations conform to biological plausibility. In addition, dietary information provided by the adolescents in the present study was based on the frequency of consumption of food items without much regard to quantity or portion size. This could have influenced the information relating to dietary habits and their associations with other variables.

## Conclusions

The high prevalence of sedentary behaviors, physical inactivity and unhealthy dietary habits among Saudi adolescents is a major public health concern. Adolescent females in particular are at great risk of sedentary behaviors and physical inactivity. Furthermore, findings from the present study confirm that unhealthy behaviors, such as increased screen time and unhealthy dietary habits, appear to aggregate among adolescents. The promotion of healthy lifestyles should be a national public health priority. In addition, there is an urgent need for national policy promoting active living and healthy eating while reducing sedentary behaviors among Saudi children and adolescents. Future research needs to address the determinants of sedentary behaviors, physical activity and inactivity, and unhealthy dietary habits and initiate interventional programs to combat unhealthy lifestyle habits among children and adolescents in Saudi Arabia.

## List of abbreviations

AAP: American Academy of Pediatrics; ATLS: Arab Teens Lifestyle Study; BMI: body mass index; IOTF: International Obesity Task Force; MET: metabolic equivalent; TV: television; WC: waist circumference; WHO: World Health Organization.

## Competing interests

The authors declare that they have no competing interests.

## Authors' contributions

**HMA **conceptualized the study and designed the research protocol, developed the questionnaire and directed all aspects of this research, including supervising data collection in Riyadh, performing the data analysis and drafting the manuscript. **NAA **directed the data collection in Al-Khobar and contributed to the writing of the manuscript. **HIA **was involved in training the female research assistants, supervised the data collection in the female schools in Riyadh and contributed to the writing of the manuscript. **DMQ **directed the data collection for the male participants in Jeddah and contributed to the writing of the manuscript. **AOM **was involved in the conception of the project, participated in the development of the dietary habits questionnaire and contributed to the writing of the manuscript. All authors critically read and approved the final version of the manuscript.
